# Direct assessment of 3D foot bone kinematics using biplanar X-ray fluoroscopy and an automatic model registration method

**DOI:** 10.1186/s13047-015-0079-4

**Published:** 2015-06-10

**Authors:** Kohta Ito, Koh Hosoda, Masahiro Shimizu, Shuhei Ikemoto, Shinnosuke Kume, Takeo Nagura, Nobuaki Imanishi, Sadakazu Aiso, Masahiro Jinzaki, Naomichi Ogihara

**Affiliations:** Department of Mechanical Engineering, Faculty of Science and Technology, Keio University, 3-14-1 Hiyoshi, Kohoku-ku Yokohama, 223-8522 Japan; Graduate School of Engineering Science, Osaka University, Toyonaka, 560-8531 Japan; School of Medicine, Keio University, Tokyo, 160-8582 Japan

## Abstract

**Background:**

Quantifying detailed 3-dimensional (3D) kinematics of the foot in contact with the ground during locomotion is crucial for understanding the biomechanical functions of the complex musculoskeletal structure of the foot. Biplanar X-ray fluoroscopic systems and model-based registration techniques have recently been employed to capture and visualise 3D foot bone movements *in vivo*, but such techniques have generally been performed manually. In the present study, we developed an automatic model-registration method with biplanar fluoroscopy for accurate measurement of 3D movements of the skeletal foot.

**Methods:**

Three-dimensional surface models of foot bones were generated prior to motion measurement based on computed tomography. The bone models generated were then registered to biplanar fluoroscopic images in a frame-by-frame manner using an optimisation technique, to maximise similarity measures between occluding contours of the bone surface models with edge-enhanced fluoroscopic images, while avoiding mutual penetration of bones. A template-matching method was also introduced to estimate the amount of bone translation and rotation prior to automatic registration.

**Results:**

We analysed 3D skeletal movements of a cadaver foot mobilized by a robotic gait simulator. The 3D kinematics of the calcaneus, talus, navicular and cuboid in the stance phase of the gait were successfully reconstructed and quantified using the proposed model-registration method. The accuracy of bone registration was evaluated as 0.27 ± 0.19 mm and 0.24 ± 0.19° (mean ± standard deviation) in translation and rotation, respectively, under static conditions, and 0.36 ± 0.19 mm and 0.42 ± 0.30° in translation and rotation, respectively, under dynamic conditions.

**Conclusions:**

The measurement was confirmed to be sufficiently accurate for actual analysis of foot kinematics. The proposed method may serve as an effective tool for understanding the biomechanical function of the human foot during locomotion.

## Background

Human bipedal locomotion is a mechanical phenomenon that transfers the body centre of mass by generating appropriate ground reaction forces. Therefore, quantifying the detailed 3-dimensional (3D) kinematics of the foot in contact with the ground during human bipedal locomotion is crucial for improving our understanding of the biomechanical functions of the complex foot musculoskeletal structures in the generation of stable and efficient bipedal locomotion. Many studies have been conducted to capture 3D kinematics of the foot bones during human walking, mainly using marker-based motion capture systems [1, 2]. However, such quantification is less accurate because of skin marker artefacts [3–6]. The use of bone pins allows direct measurement of 3D bone kinematics, but is very invasive [4–8]. Accurate measurement of the 3D kinematics of foot skeletal movement during locomotion thus remains a challenging problem.

Radiographic techniques provide a more direct, but less-invasive way to measure bone kinematics. Kinematic analyses of skeletal motions using single or biplanar X-ray fluoroscopic systems have recently become widespread in the field of biomechanics, yielding significant results. For example, dynamic kinematic measurements of the human knee [9], shoulder [10] and spine [11] have been conducted using X-ray fluoroscopy to capture precise joint kinematics *in vivo*. Furthermore, such techniques have been applied to the analysis of animal locomotion, bringing substantial benefits to uncovering morphofunctional relationships between animal anatomy and locomotion [12, 13]. In the same vein, X-ray fluoroscopic systems have recently been applied to analyses of 3D foot and ankle kinematics *in vivo*. For example, Yamaguchi et al. [14] and Fukano et al. [15] have employed single-plane fluoroscopy and Wan et al. [16], de Asla et al. [17], and Kozanek et al. [18] have used biplanar fluoroscopy to successfully reconstruct 3D kinematics of the ankle and subtalar joints *in vivo*.

In many of these foot studies, however, 3D surface models of the bones are manually matched to fluoroscopic images, by manipulating each bone in a virtual space to be positioned and rotated using a computer mouse. Manual registration is indeed challenging and time-consuming work that requires extreme patience, possibly resulting in inaccuracy of the reconstructed positions and orientations of the bones. The potential for inaccuracy in model registration may be relatively larger in the case of foot analyses, since bone registration is more difficult because the foot skeleton consists of 27 small, overlapping bones. Although many automatic registration methods have been proposed for relatively simple skeletal structures such as the knee [19–21], hip [22], shoulder [23] and spinal vertebrae [24], no studies have attempted to develop an accurate registration method for foot bones. Very recently, Campbell et al. [25] reported automatic registration of the tibia, fibula and calcaneus in walking, but did not include foot bones other than the calcaneus.

In the present study, we developed an automatic model-registration method with biplanar fluoroscopy for accurate measurement of 3D foot skeletal movements. To evaluate the feasibility and validity of the proposed method, we analysed 3D skeletal movements of a cadaver foot mobilized by the robotic gait simulator using a biplanar X-ray fluoroscopy system and a proposed model-registration method.

## Methods

**Fig. 1:**
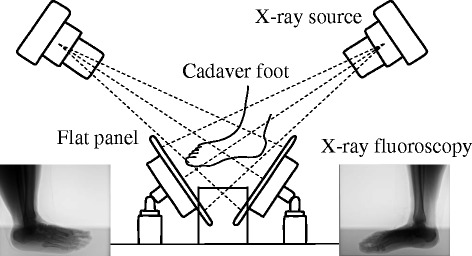
Biplanar X-ray fluoroscopy system. The system consists of two X-ray sources and corresponding detector panels positioned in a quasi-orthogonal arrangement

In this study, we attempted to register a 3D surface model of foot bones with two fluoroscopic images to calculate temporal changes in the positions and orientations of these bones in the 3D space. For this, we used a custom-made X-ray biplanar fluoroscopy system, consisting of two sets of X-ray sources and flat panels positioned in a quasi-orthogonal arrangement (Shimadzu, Kyoto, Japan) (Fig. 1). The flat panels consist of a precision amorphous silicon metal-insulator semiconductor sensor and thin-film transistor array with a field size of 17 inches × 14 inches and a detector matrix of 2688 × 2208 pixels. Anode heat storage capacity and the focus size of the X-ray tube were 400 kHU and 0.3/0.8 mm, respectively. A series of paired dynamic fluoroscopic images can be acquired at 15 Hz. Dimensions of the measurement volume are approximately 30 cm × 20 cm × 20 cm in the present setting.

To reconstruct 3D movements of the foot bones from the 2D images, spatial calibration of the biplanar fluoroscopic system is necessary. We used two calibration objects with spherical metal markers (a planar L-shaped structure and a linear rigid bar) to obtain the projection geometry of the biplanar fluoroscopic system based on the direct linear transformation method [26], assuming that a fluoroscopic system can be modelled as an ideal perspective projection of a point-source X-ray onto a flat panel. Correction of non-linear distortion due to the photocathode curvature of the image intensifier was not necessary in the present study, as we used flat panel detectors, but we corrected for linear trapezoidal distortion of fluoroscopic images due to imperfect orthogonality of the X-ray axis to the flat panel detector. Mean and standard deviation of the calibration error was 0.076 ± 0.058 mm in the present study.

### 3D bone surface models

To create bone surface models registered to the fluoroscopic images, a cadaver foot was scanned using a medical computed tomography (CT) scanner (Aquilion ONE; Toshiba Medical Systems,　Japan), and cross-sectional images were reconstructed at 0.25-mm intervals, with a pixel size of 0.316 mm. Images were then transferred to medical imaging software (Analyze version 9.0; Mayo Clinic, USA) for segmentation of the foot bones. Surface models of the bones were generated as triangular polygonal models by the marching cube method. The polygonal mesh models were then regenerated after polygon reduction using reverse-engineering software (RapidForm 2006; INUS Technology, Korea). A local coordinate system was created to describe the relative positions and orientations of each of the four tarsal bones, i.e., calcaneus, talus, cuboid, and navicular. Specifically, three or four landmarks were defined on each of the bones and an orthogonal coordinate system was calculated [27]. The x-, y- and z-axes in the present study approximately correspond to the anteroposterior, mediolateral, and dorsoplantar directions, respectively. Origins of the coordinate systems were defined at the centroid of each polygonal mesh bone model. We used y-x-z Euler angles to describe the absolute and relative orientations of a bone, with *ϕ*, *θ* and *ψ* as the rotational angles around the y (plantarflexion-dorsiflexion), x (inversion-eversion), and z (adduction-abduction) axes, respectively.

### Automatic model-registration method

**Fig. 2:**
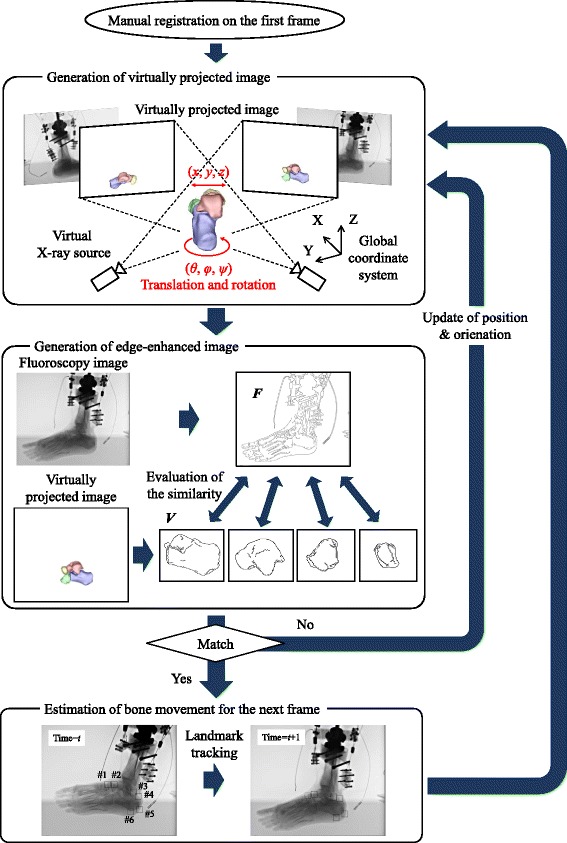
Outline of the registration method. The initial position of the bone model on the first frame is defined manually, and virtually projected images are generated for the bone models. Edge-enhanced fluoroscopic images and virtually projected images, *F* and *V*, are obtained, and the positions and orientations of the bones are calculated based on the similarity measure (Equation 1). Template-matching is used to obtain the initial guess of bone positions and orientations on the next frame, and the registration proceeds in a consecutive manner

An outline of the model-matching method is illustrated in Fig. 2. To register the bone model to the fluoroscopy, we reproduced the projection geometry of the fluoroscopy system in a virtual space using the calibration result. We searched for the position and orientation of the bone model in which the virtual projection image becomes congruent with the two fluoroscopic images using an edge-matching algorithm [21]. First, the Gaussian filter was applied to the fluoroscopic images for noise reduction. The Canny filter was then used to obtain edge-enhanced fluoroscopic and virtually projected images, *F* and *V*, respectively. High-and low-threshold values of the Canny filter were set to 30 and 20 for the left, 20 and 10 for the right, respectively, to optimise edge-enhancement. For the virtually projected images, high- and low-threshold values were set to 300 and 200, respectively. Edge segments longer than 100 pixels in *V* and 10 pixels in *F* were considered informative and selected for further calculation. To create an intensity gradient along the edges, the edge-enhanced virtually projected image *V* was dilated twice and the dilated-edge image *V*’ was obtained. Such image processing was conducted by an open-source computer vision library, OpenCV version 2.1. The position (*x*, *y*, *z*) and orientation (*θ, ϕ, ψ*) of the bone model that best matched the two fluoroscopic images were calculated by maximising the similarity measure *S*, defined as:

1 $$ {S}_i=\frac{F_i^L\cdot {V}_i^L\hbox{'}}{\sqrt{F_i^L\cdot {F}_i^L}\sqrt{V_i^L\hbox{'}\cdot {V}_i^L\hbox{'}}}+\frac{F_i^R\cdot {V}_i^R\hbox{'}}{\sqrt{F_i^R\cdot {F}_i^R}\sqrt{V_i^R\hbox{'}\cdot {V}_i^R\hbox{'}}} $$

where subscript *i* represents the bone number, and subscripts *L* and *R* denote left and right images, respectively. This similarity measure represents a summation of correlation coefficients between the two edge-enhanced images of the left and right fluoroscopic systems.

If more than two bones were registered, we checked for collision and penetration of registered bone models. The position and orientation of the bone model can thus be calculated by maximising the following objective function *I*:

2 $$ I={\displaystyle \sum_i\left({S}_i-{\displaystyle \sum_j\mu {D}_{ij}}\right)}\to Max $$

where *D*_*ij*_ is the penetration depth between the *i*th and *j*th bones, and *μ* is the coefficient (=1000). The penalty function was calculated by a collision-detection library (SmartCollision version 2.3; I-NET Corporation, Japan), which can efficiently detect collisions and calculate penetration depths between 3D mesh models. We used a quasi-Newtonian method for optimisation, and the calculation was conducted with custom-made software using Microsoft Visual Studio C++ 2010. Due to the complexity of the edge-enhanced images from foot fluoroscopy, the optimisation function involves numerous local minima. In the present study, therefore, partial derivatives were computed with multiple different step sizes for this gradient-based optimisation algorithm.

The initial position of the bone model on the first frame was defined manually. Automatic registration was then performed to search for the true position and orientation of the bones on the first frame. This result was used as the initial guess for optimisation on the next frame and registration proceeds one after another. However, if movement of the bone is relatively fast compared to the frame rate of the fluoroscopic system, bone translation and rotation in two consecutive frames might be quite large, possibly resulting in tracking failure. Prior to the automatic registration, therefore, we roughly estimated the amount of bone translation and rotation in two consecutive frames using a template-matching method [28]. Specifically, small areas (30 × 30 pixels) corresponding to bony landmarks were defined manually on the first frame as template images, and corresponding matching areas were automatically identified by comparing template images against the subsequent image based on cross-correlation. The same set of bony landmarks was traced on both fluoroscopic images. The 3D positions of bony landmarks were calculated based on triangulation for estimation of initial guesses for the position and orientation of each bone. Template images were updated for template-matching of the next frame.

### Accuracy-evaluation experiment

To evaluate the accuracy of the proposed registration method, we used four articulated dry tarsal bones with four spherical metal markers attached to each bone. We placed the articulated tarsal bones in the fluoroscopic system in five different positions and orientations, and corresponding fluoroscopic images were obtained. We solved for the positions and orientations of each bone in two ways, one by the proposed registration method and the other using the 3D positions of the four markers calculated based on calibrated projection geometry. The positions and orientations of the bones calculated by the latter were taken as true values and the registration errors were evaluated (static evaluation). We also used a single unarticulated talus and calcaneus to see how the overlap of articulated tarsal bones in fluoroscopic images affects registration performance. In addition, we conducted the same evaluation experiment while manually translating and rotating the articulated tarsal bones to evaluate how movements affect registration performance.

The dry tarsal bones used in this experimental evaluation were obtained from the Laboratory of Physical Anthropology at Kyoto University, and were scanned using a medical CT scanner. Cross-sectional images were reconstructed at 0.5-mm intervals, with a pixel size of 0.234 mm. The bone models were constructed as described previously.

### Cadaver experiment

The present study reconstructed the dynamic skeletal movements of a cadaver foot (male, 78 years old) mobilized by a robotic gait simulator to evaluate how well the proposed method can track and register foot bones in motion during the stance phase of bipedal walking. The cadaver used in our study was donated to the Clinical Anatomy Laboratory at Keio University School of Medicine with the consent of the family. The present study was approved by the ethics committee of the School of Medicine and the Faculty of Science and Technology at Keio University.

Briefly, the simulator has three legs (fore, middle and hind legs), with the cadaver foot fixed to the middle leg arranged radially in the sagittal plane. The middle leg replicates the movements of the cadaver foot from heel-contact to toe-off, while the hind and fore legs replicate toe-off and heel-contact of the contralateral limb, respectively, so the X-ray image is not interrupted by the swing leg. The simulator initially stands only on the hind leg, and is then released to fall forward so that the middle cadaver foot makes heel contact. The simulator continuously rotates forward so that the fore leg makes the foot-ground contact and the cadaver foot finally makes toe-off. Tendons of the tibialis anterior and soleus were connected to pneumatic actuators to apply forces at the appropriate moment to reproduce how the foot would function during walking. Development of the robotic gait simulator will be described elsewhere.

Using fluoroscopic images of this cadaveric walking, we quantified 3D skeletal movements of the calcaneus, talus, navicular and cuboid in the stance phase of the gait and evaluated system performance. Template images were selected at the following six positions: 1) most anterodorsal point of the navicular; 2) most anterodorsal point of the talus; 3) most posterior point of the talus; 4) most superior point; 5) most posterior point; and 6) most inferior point of the calcaneal tuberosity (Fig. 2) for this analysis.

## Results

Registration errors of the four bones in the static evaluation are summarised in Table 1. Means and standard deviations of the registration errors of the articulated bones were 0.27 ± 0.19 mm in translation and 0.24 ± 0.19° in rotation. Errors were relatively larger for the navicular and cuboid (0.33 ± 0.22 mm in translation and 0.27 ± 0.20° in rotation). Registration errors of the unarticulated talus and calcaneus were 0.15 ± 0.12 mm in translation and 0.14 ± 0.13° in rotation (mean ± standard deviation), indicating that errors were relatively larger in the articulated bones.

**Table 1:** Mean (standard deviation) errors of articulated bones and unarticulated bones in static evaluation

	Articulated bones				Unarticulated bones	
	Calcaneus	Talus	Cuboid	Navicular	Calcaneus	Talus
*dx* (mm)	0.28 (0.12)	0.14 (0.06)	0.40 (0.20)	0.39 (0.33)	0.11 (0.08)	0.15 (0.05)
*dy* (mm)	0.17 (0.10)	0.24 (0.18)	0.19 (0.09)	0.37 (0.21)	0.17 (0.07)	0.20 (0.19)
*dz* (mm)	0.22 (0.10)	0.21 (0.13)	0.30 (0.19)	0.33 (0.15)	0.09 (0.07)	0.16 (0.08)
*θ* (deg)	0.17 (0.08)	0.16 (0.07)	0.22 (0.11)	0.29 (0.20)	0.08 (0.07)	0.15 (0.09)
*ϕ* (deg)	0.20 (0.20)	0.21 (0.23)	0.19 (0.21)	0.23 (0.16)	0.16 (0.13)	0.20 (0.12)
*ψ* (deg)	0.18 (0.12)	0.29 (0.22)	0.39 (0.27)	0.31 (0.15)	0.15 (0.14)	0.13 (0.11)

Registration errors of the four bones in the dynamic evaluation are summarised in Table 2. Means and standard deviations of the registration errors were 0.36 ± 0.19 mm in translation and 0.42 ± 0.30° in rotation, and those of the unarticulated bones were 0.22 ± 0.13 mm in translation and 0.28 ± 0.21° in rotation. Errors were relatively larger in the dynamic evaluation.

**Table 2:** Mean (standard deviation) errors of articulated bones and unarticulated bones in dynamic evaluation

	Articulated bones				Unarticulated bones	
	Calcaneus	Talus	Cuboid	Navicular	Calcaneus	Talus
*dx* (mm)	0.17 (0.16)	0.46 (0.25)	0.51 (0.22)	0.28 (0.17)	0.14 (0.06)	0.20 (0.11)
*dy* (mm)	0.40 (0.22)	0.23 (0.08)	0.40 (0.22)	0.62 (0.23)	0.42 (0.13)	0.18 (0.08)
*dz* (mm)	0.34 (0.24)	0.38 (0.10)	0.32 (0.17)	0.22 (0.13)	0.17 (0.08)	0.23 (0.06)
*θ* (deg)	0.33 (0.28)	0.45 (0.29)	0.29 (0.19)	0.56 (0.29)	0.29 (0.17)	0.36 (0.28)
*ϕ* (deg)	0.40 (0.30)	0.46 (0.28)	0.46 (0.40)	0.56 (0.38)	0.25 (0.26)	0.28 (0.16)
*ψ* (deg)	0.25 (0.21)	0.41 (0.31)	0.43 (0.29)	0.37 (0.23)	0.24 (0.14)	0.28 (0.17)

**Fig. 3:**
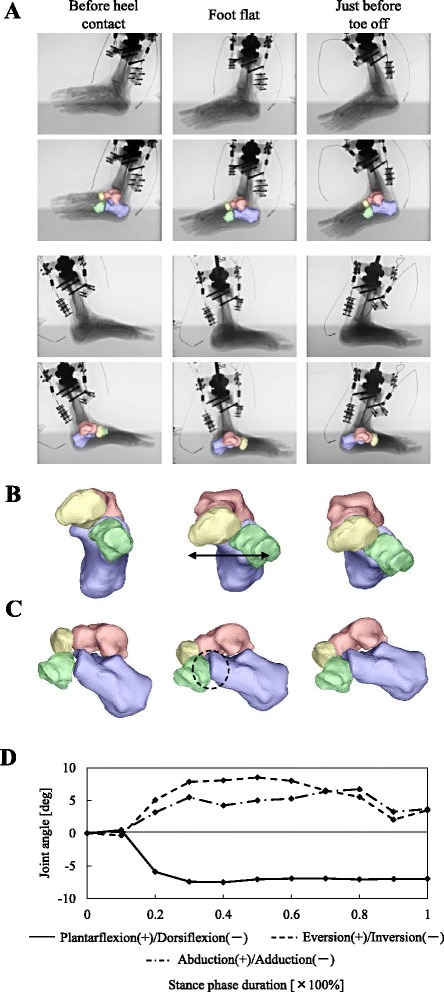
Three-dimensional kinematics of the cadaver foot mobilized by a robotic simulator. **a)** Biplanar X-ray fluoroscopic images and corresponding registration results. Calcaneus, talus, navicular and cuboid at just before heel-contact, foot flat and just before toe-off are presented. **b, c)** The 3D movements of the tarsal bones are displayed in sagittal and anterior views, respectively. After heel contact, the cuboid and calcaneus are observed to move laterally away from the navicular. The articular surface of the cubocalcaneal joint is closely matched during the foot-flat. **d)** Tri-axial rotational angles of the calcaneus with respect to the talus. Angles are defined as zero at the time of heel-contact. Positive angles represent abduction, eversion, and plantarflexion of the subtalar joint

Registration results of the cadaver foot mobilized by the simulator are shown in Fig. 3a. Surface models of the four tarsal bones were successfully matched with the corresponding fluoroscopic images. Figure 3b and c present anterior and sagittal views of the reconstructed foot skeletal movement. After heel contact, the cuboid and calcaneus were observed to move laterally away from the navicular (Fig. 3b) and this lateral shift of the cuboid was maintained until toe-off. The cuboid articular surface of the calcaneus and the posterior articular surface of the cuboid were also noted to be closely matched to each other to form a closed, packed position of the two articular facets during the foot-flat period (Fig. 3c). Fig. 3d shows temporal changes in the subtalar joints during the stance phase of cadaver foot walking. After heel contact, the calcaneus was found to be dorsiflexed, everted and abducted with respect to the talus. In the late stance phase, however, the calcaneus was slightly inverted and adducted.

**Fig. 4:**
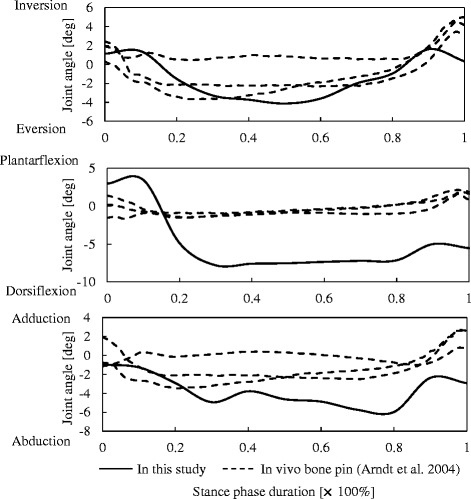
Comparisons of subtalar joint angles between the cadaver foot mobilized by a robotic simulator and those measured *in vivo* using intracortical pins during the stance phase of walking [7]. Solid line = cadaver foot. Dotted lines = actual human walking (3 subjects) [7]

The subtalar kinematics of the cadaver foot quantified using the proposed model-matching method were compared with those measured *in vivo* using intracortical pins during the stance phase of walking [7] (Fig. 4). For this comparisons, subtalar joint angle profiles in Fig. 3d were recalculated based on bone coordinate systems defined in the literature [7]. Since the rotational sequences of Euler angles were not presented in the literature, we assumed that the sequence was the same as the one we defined, confirming that the rotational sequence did not greatly affect the joint angle profile, as subtalar joint rotation is relatively small. Here the subtalar joint angles are positive for inversion, plantarflexion, and adduction. Inversion-eversion joint angle profile was similar between the present in vitro and the reported *in vivo* studies (Fig. 4). However, the range of joint motion for plantar-dorsiflexion was found to be much larger in the cadaver foot. Furthermore, although the calcaneus was gradually adducted with respect to the talus in the late stance phase in human walking, this bone was relatively in a more abducted position in the present cadaver study.

## Discussion

In the present study, we developed an automatic model-registration method with biplanar fluoroscopy for accurate measurement of 3D foot skeletal movements, and demonstrated that 3D kinematics of the calcaneus, talus, navicular and cuboid in the stance phase of gait were successfully reconstructed using the proposed method. In this study, we used the similarity measure (Equation 1) for the registration of bone models with fluoroscopic images. However, the similarity measure alone did not produce satisfactory results, since foot bones overlapped heavily with one another on the fluoroscopic images. Furthermore, registration failed when bone translation and rotation in two consecutive frames were large. We therefore introduced the penetration penalty function, landmark tracking based on template matching, and partial derivatives of the objective function with multiple step sizes for our registration process. With these efforts, automatic tracking of the positions and orientations of foot bones from the two fluoroscopic images become possible as presented in Fig. 3.

The accuracy of foot bone registration was confirmed to be 0.27 ± 0.19 mm in translation and 0.24 ± 0.19° in rotation in the static condition, and 0.36 ± 0.19 mm in translation and 0.42 ± 0.30° in rotation in the dynamic condition. The registration error of published automatic model-registration methods was reportedly 0.16-0.33 mm and 0.16-0.58° for translation and rotation, respectively [20, 22, 23], indicating that similar levels of accuracy were obtained using the proposed method. In those previous studies, however, registrations were performed on relatively simple skeletal structures such as knee, hip and shoulder joints, where there is very little overlap between bones. Furthermore, we tried herein to measure movements of small foot bones such as the navicular and cuboid in which relatively few characteristic morphological features could be detected. We therefore believe that the accuracy of the proposed method should be evaluated as relatively good. Based on kinematic measurements using bone pins, the range of subtalar joint motion is reported to be about 2.5-3.8 mm in translation and 2.8-9.7° in rotation in cadaveric studies [29–31] during human walking, and about 3.3-9.8° from *in vivo* studies [6–8]. Range of motion of tarsometatarsal joints has been reported as about 4.6-12.9° in cadaveric studies [30, 31], and 4.1-13.3° from *in vivo* studies [6–8]. The present registration method should therefore be considered as sufficiently accurate for quantitative description of the talar and tarsometatarsal joint kinematics. It must be noted, however, that evaluation of accuracy using dry bones has probably underestimated the actual error, as bone edges are less distinct when surrounded by soft tissues. This should be confirmed in future studies.

Manual registration requires a great deal of patience, particularly if there are many small bones to be registered as in the case of the foot. On the other hand, the present study allows automatic registration of multiple bones while possibly eliminating some of the errors associated with the tediousness of manual registration. The accuracy of the biplanar manual registration is reportedly about 0.3-0.4 mm in translation and 0.4-0.5° in rotation [10, 11]. Consequently, the present framework may provide useful support for clarifying 3D kinematics of the foot in contact with the ground during human bipedal locomotion.

Recently, a digitally reconstructed radiograph (DRR) method has frequently been applied to assess skeletal movements from fluoroscopic images, as the DRR method offers more information for matching CT volumes to fluoroscopic images, since it can use internal bone morphology such as cortical thickness [9, 22, 25, 32]. Although the DRR method probably requires more processing time than the present contour-matching method, the DRR certainly provides more information than contours and theoretically yields better performance. Miranda et al. [32] recently proposed DRR-based tracking software using GPU acceleration and computational time is decreasing. Incorporating the DRR method in the present study may be an option in future studies to achieve better registration results.

However, the proposed method has some limitations. One is that the computational cost could become very high if the number of bones to be registered is large. Ideally, the positions and orientations of all 27 foot bones should be registered simultaneously. However, it is probably more practical to perform calculations in a step-by-step manner for simplicity, such as calculating the positions and orientations of the four bones as the first step, then proceeding to the metatarsals, cuneiforms, tibia, and so on, to reconstruct 3D skeletal movements of the entire foot. Another limitation is that the present registration process is not fully automatic, because the initial guess for the positions and orientations of bones on the first frame and the bony landmarks to be tracked should be determined manually. A simple method to place bone models in a vicinity of the true positions and orientations on the first frame seems necessary. For example, Haase et al. [33] proposed automatic annotation of landmarks in biplanar fluoroscopy based on active appearance models. Applying such a method may be useful for placing bones in the vicinity of the true position and orientation on the first frame. Lastly, although the present method can be applied to quantify foot skeletal movements *in vivo*, we quantified 3D skeletal movements of a cadaver foot mobilized by a robotic gait simulator using the proposed method. However, as shown in Fig. 4, reconstructed subtalar joint kinematics are currently not in good agreement with those of actual human walking as reported in the literature, mainly due to difficulties associated with the robotic gait simulator. More work is clearly needed to mobilize the cadaver foot in a manner similar to the way the human foot actually moves and mechanically interacts with the ground, to further elucidate the biomechanical functions of the complex musculoskeletal structures of the human foot during bipedal locomotion.

## Conclusions

In the present study, we developed an automatic model-registration method with biplanar fluoroscopy for accurate measurement of 3D movements of the skeletal foot. Specifically, 3D surface models of foot bones were generated prior to motion measurement based on computed tomography, and the bone models generated were then registered to biplanar fluoroscopic images in a frame-by-frame manner to maximise similarity measures between occluding contours of the bone surface models with edge-enhanced fluoroscopic images, while avoiding mutual penetration of bones. Measurement was confirmed to be sufficiently accurate for actual analysis of foot kinematics. The proposed method may serve as an effective tool for understanding the biomechanical function of the human foot during locomotion.
